# Bullying, Cyberbullying, Anxiety, and Depression in a Sample of Youth during the Coronavirus Pandemic

**DOI:** 10.3390/pediatric13030064

**Published:** 2021-09-14

**Authors:** Elizabeth Englander

**Affiliations:** Massachusetts Aggression Reduction Center, Bridgewater State University, Bridgewater, MA 02325, USA; eenglander@bridgew.edu

**Keywords:** bullying, cyberbullying, pandemic, social and emotional learning, depression, anxiety

## Abstract

While we know that the pandemic and its social isolation, loss of school experiences, increased screen use, and financial stress have likely had a psychological impact upon children and teens, little research has been done directly with youth to assess social and emotional factors during the pandemic and in its immediate aftermath. In this study, a sample of 240 youth reported on their experiences with bullying, fighting, sexting, cyberbullying, anxiety, and depression during the period from March 2020 to April 2021. The results indicated that bullying, cyberbullying, sexting, and fighting showed only small or no increases, but anxiety and depression were dramatically increased relative to before the pandemic. Female and LGBTQ youth were particularly vulnerable during the months since March 2020. The results suggest that youth will need positive social experiences and, in some cases, psychological interventions and treatment to restore emotional equilibrium in the months ahead.

The COVID-19 virus began to spread in the United States in earnest around the middle of March 2020. Although the first cases were reported in the United States in February, there were cases in all 50 states by mid-March, according to the CDC. (Available online: https://www.cdc.gov/mmwr/volumes/69/wr/mm6915e4.htm#:~:text=Community%20transmission%20of%20COVID%2D,of%20COVID%2D19 (accessed on 1 June 2021)). As the country shut down in an attempt to control the spread of the virus, large-scale job layoffs also began in March, although some economists pegged the recession as truly starting during the first bloom of the coronavirus in February. (Available online: https://www.marketwatch.com/story/the-nber-is-wrong-the-recession-began-in-march-not-february-2020-06-08 (accessed on 1 June 2021)). Although children are typically not primary breadwinners in their home, financial stress can affect them in multiple ways. Some children lost their homes; an unknown proportion had parents or caregivers who have lost their jobs. Parental stress trickles down to children, who may or may not know details, but who are acutely sensitive to the emotional state of their caregivers. It is also significant that this financial stress was not a slow, predictable slide; instead, it was more sudden and potentially traumatic, like falling off a cliff.

Pandemic and recession were not the only social changes experienced by children. On 26 May, after many weeks of social isolation, the American people watched brutal cell phone footage, released on social media, of the murder of George Floyd the day before, determined by a trial to be at the hands of Minneapolis police. This case sparked a national movement seeking to address police violence and the profound racism that still permeates the United States, both legally and culturally. It is important to note that the social unrest that begun in May 2020 is substantially unlike the pandemic and the recession, in the sense that it seeks to redress a profound wrong and, thereby, improve our society. However, all three events represent upheaval and change, and thus all three may have affected children emotionally.

The primary source of anxiety and distress for children may have been (or may still be) the coronavirus itself. Some of the research that has been conducted suggests that simply the existence of the virus poses a source of distress for children (and for adults as well, it must be said) [1]. This….“thing”….that is out there, and that might hurt you, certainly sounds like many of the vague childhood fears that you may recall from your own youth. Of course, the virus also sickened and killed more than 600,000 Americans. Some youth have lost family members or seen them become ill from COVID-19.

The virus also posed a third source of stress and difficulty for children, namely, it necessitated profound social isolation for them and their families. In an effort to control the spread of the virus, children and their families were often alone at home. Unable to go to school or play with peers, children may have been particularly vulnerable to the stressors that social isolation also wrought on their parents and families. The destructive nature of this social isolation, and keeping children from school, is acknowledged, but most experts believed that it was still preferable to the spread of the virus. However, some child development experts have questioned if closing schools poses a greater risk for children than exposure to COVID-19, because of the apparently increased possibility of child abuse or other at-home traumatic incidents. (Available online: https://www.cnn.com/videos/tv/2020/11/20/lead-dr-christakis-live-jake-tapper.cnn (accessed on 1 June 2021)).

## 1. Children’s Mental Health during the Pandemic

There has been some research that has given us some insight into how the last few months have affected children psychologically and emotionally. One study of 1784 Chinese children in the Hubei province [2], which includes Wuhan, found that elevated numbers of children expressed depressive symptoms and anxiety, in comparison with Chinese children studied before the pandemic began. The study was also interesting because it compared children in Wuhan to children in Huangshi, which was not as badly affected by the virus itself, but was also shut down, with social isolation. While the children in both towns were affected, the children in Wuhan reported more severe symptoms. This suggests that it is not only the social isolation that affects children, but possibly also the existence of the virus outside their home (the fear of it) and the children’s knowledge about any acquaintances or relatives who were sickened or even died as a result of the virus.

Save The Children conducted surveys in April 2020, in the United States and in Europe, examining the mental health consequences of the pandemic and recession. (Available online: https://www.savethechildren.net/news/covid-19-2-3-parents-us-worry-about-their-child%E2%80%99s-emotional-mental-well-being# (accessed on 3 June 2021)). During the lockdown, 17% of children in the surveys felt depressed *often* or *always*; 25% reported dealing with anxiety; 32% struggled to sleep; and 30% feared the COVID-19 virus. As is the case during most crises, parental stress increased and parental resources decreased. When that happens, increases in reports to child abuse and domestic abuse hotlines are almost always noted. This crisis was no exception. The National Center for Missing and Exploited Children (NCMEC) reported a huge increase in the number of abuse cases reported during April. In April 2019, approximately 1 million cases of child abuse were reported, but one year later, that number was over 4 million. (Available online: https://www.click2houston.com/news/national/2020/05/29/the-pandemic-is-causing-an-exponential-rise-in-the-online-exploitation-of-children-experts-say/ (accessed on 3 June 2021)). A Texas hospital reported a notable increase in hospital admissions involving child abuse—2 to 3 times higher than normal. (Available online: https://dfw.cbslocal.com/2020/03/20/texas-hospital-spike-severe-child-abuse-cases-coronavirus/ (accessed on 4 June 2021)). In New York, domestic violence calls were twice as common as is typically seen during this time of year. (Available online: https://www.nytimes.com/2020/05/15/us/domestic-violence-coronavirus.html (accessed on 1 June 2021)). Finally, the National Sexual Assault Hotline reported a 22% increase in monthly calls from children under the age of eighteen to the hotline; most children who called reported that the sexual abuse perpetrator was a family member with whom they were living. (Available online: https://www.npr.org/sections/coronavirus-live-updates/2020/04/28/847251985/child-sexual-abuse-reports-are-on-the-rise-amid-lockdown-orders (accessed on 31 May 2021)).

Socializing face-to-face with peers is critical for children, but during lockdowns, many children felt isolated. The ParentsTogether survey found that screen time during the pandemic had increased dramatically, doubling on average, and while much of that screen time was undoubtedly spent doing schoolwork, a great deal of it was also almost certainly socializing in the form of social media, gaming, and other interactions online.

A critical question remains: during the most severe months of the pandemic (roughly March 2020 to May 2021), did fighting, bullying, and cyberbullying increase between youth in the United States? Further, what mental health challenges did students report during the same period? Some experts predicted that, because youth spent so much time online during the pandemic, rates of cyberbullying and sexting could increase significantly. (Available online: https://www.healio.com/news/pediatrics/20200330/cyberbullying-may-increase-during-covid19-pandemic-expert-says (accessed on 31 May 2021)). However, such increases have not been widely studied.

## 2. Methods

The current study surveyed 240 youth aged 17 or older between January and April 2021. It utilized a convenience sample of university students in Massachusetts. The online survey asked students about their social experiences and mental health functioning since March 2020. Because of the pandemic, the mandatory Subject Pool linked to the Introduction to Psychology course was not functional at the University, so students who completed the survey online were all volunteers, some of whom may have earned extra credit in their classes.

The sample was composed of the following age groups: 39% were aged 17–19, 43% were aged 20–21, and 18% were 22 years or older. Of the students, 69% identified themselves as Caucasian and the remaining 31% self-identified as students of color. Moreover, 24% reported that they were members of the LGBTQ community (lesbian, gay, bisexual, transgender, queer, nonconforming).

It is important to note that bullying, cyberbullying, anxiety, and depression were not formally diagnosed in the sample. Limitations imposed by the pandemic did not permit formal diagnoses. Instead, this study examined the subjects’ self-perceived involvement in fighting, bullying, cyberbullying, depression, and anxiety. Subjects were also asked about their involvement in sexting (sending nude pictures electronically to a peer) before and during the pandemic. 

## 3. Results

Fighting, quarreling, and other offline conflict did not increase during the pandemic months. More than a third (35%) of subjects said that it remained at low levels, 23% said such conflict decreased, and 42% reported that conflict was high before the pandemic and remained high during the months since March 2020. Subjects who reported that conflict stayed low before and during the pandemic also tended to report lower levels of anxiety and depression; the differences approached significance (X^2^ = 4.929(2), *p* < 0.085). Similarly, only 8% of subjects reported increases in traditional (in person) bullying, 35% said levels of bullying remained low, 21% reported that levels of bullying remained high, and 36% reported decreases in bullying. Different levels and changes in bullying status were not associated with increased anxiety or depression.

Almost two-thirds of subjects reported some increased use of social media during the pandemic. Females were slightly more likely than males to report this (71% vs. 57%), but problematic digital behaviors did not increase correspondingly. Digital forms of fighting and bullying (“cyberbullying”) rose slightly more than traditional bullying; 11% of subjects reported that these had increased since March 2020. Similarly, 11% reported a decrease, and most subjects reported no change. Further, 28% said that cyberbullying had been frequent before the pandemic and remained so, while 50% reported that it had been infrequent before the pandemic, and was also infrequent in the months since March 2020. The subjects who described cyberbullying as having either increased during the pandemic or remained frequent showed the most anxiety and depression, although the differences approached significance (X^2^ = 6.646(3), *p* < 0.084). 

Another digital behavior, “sexting”, showed very little change; only 2% reported that it increased in frequency; while another 3% reported that it decreased in frequency; and 47% and 48% reported that it remained infrequent or frequent, respectively. Changes in sexting behavior were not associated with increased anxiety and depression.

Increases in social media use were primarily reported for positive engagement, such as connecting with others (63%) and entertainment (46%). Subjects who increased their SM use for connecting with others reported less anxiety and depression (67% vs. 33%, X^2^ = 3.621(1), *p* < 0.05). A total of 23% of subjects said they were gaming more often than before March 2020, but increases in gaming were not associated with anxiety and depression. The majority of subjects who reported increases in entertainment use of the Internet said they were watching short videos (e.g., on YouTube) or television shows or movies (83% and 73%, respectively).

Mental health showed much more dramatic changes associated with stressors and loss during the pandemic. Among the subjects, 68% reported that they missed seeing people during the pandemic, and subjects who missed seeing people were significantly more likely to also report increased anxiety and depression (76% vs. 51%, X^2^ = 13.8(1), *p* < 0.000). Further, 31% reported that they felt isolated since March 2020, and these subjects were significantly more likely to report increased anxiety and depression as well (36% vs. 20%, X^2^ = 5.752(1), *p* < 0.016). Interestingly, about 17% reported that they did not mind staying home and 12% reported that they remained just as social as they had been before the pandemic. Subjects who reported that they preferred staying home to going out reported significantly lower levels of anxiety and depression (X^2^ = 18.65, *p* < 0.005). Subjects also reported that the loss of opportunities was the single greatest source of stress, referring specifically to losing school and work opportunities. Moreover, 38% reported that having someone close to them become sick with COVID-19 was a significant source of stress; 16% reported that they tested positive, but did not become ill, and 15% reported that someone close to them had died. School opportunity losses were significantly associated with increased anxiety and depression (63% vs. 43%, X^2^ = 8.146(1), *p* < 0.004), as was having someone close to them become sick with COVID-19 (43% vs. 28%, X^2^ = 4.296(1), *p* < 0.038). Other stressors were not.

An astonishing 71% of subjects reported increases in anxiety, depression, or both. The largest group of these, 42%, reported that they felt that they were experiencing significantly higher rates of *both* anxiety and depression since March 2020. Almost one-quarter (22%) reported that only their anxiety had increased, and 7% reported that only depression had increased. White students and students of color reported similar levels of anxiety and depression (X^2^ = 0.16(1), *p* = n.s.). Males were less likely to report increased anxiety or depression than females, but almost all LGBTQ subjects (96%) reported perceived increases in anxiety and depression (X^2^ = 5.703(1), *p* < 0.01).

## 4. Discussion

Despite some predictions to the contrary, this survey did not find dramatic increases in social problems such as fighting and bullying in the period between March 2020 and April 2021. Even digital forms of problematic behaviors, such as cyberbullying and sexting, showed relatively minor increases. Traditional interpersonal conflict that occurs in person, that is, bullying and fighting, may have failed to increase simply because of less opportunity; students were kept out of school and home for many of the months in question, and social distancing was largely enforced in Massachusetts, where the subjects were surveyed. The generally stable rates of cyberbullying and sexting are harder to explain, given the dramatic increases in screen use by virtually all youth. One possible explanation is that, possibly, the dramatic nature of the social changes undergone by youth has made aggression with peers (whom they miss seeing) less attractive. It is also possible that interactions with peers who were not friends decreased during the social distancing periods. Finally, the nature of this sample (17 years or older) may account for the dearth of findings; perhaps, cyberbullying increased among younger children but not among older teens and young adults simply because of maturation and the extreme social circumstances, of which older teens would have been more conscious.

In contrast to the small rises in bullying and cyberbullying, self-reported increases in anxiety and depression were extremely common, especially for females and for LGBTQ subjects (who are historically more vulnerable). Financial stressors, being confined for extended periods of time with family, missing friends and other close social relationships, and similar factors could certainly account for anxiety and depression. It is generally acknowledged that women bore the brunt of having to home-school children while attempting to keep working:

*While all women have been impacted, three major groups have experienced some of the largest challenges: working mothers, women in senior management positions, and Black women. This disparity came across as particularly stark with parents of kids under ten: the rate at which women in this group were considering leaving was ten percentage points higher than for men. And women in heterosexual dual-career couples who have children also reported larger increases in their time spent on household responsibilities since the pandemic began*. Available online: https://www.mckinsey.com/featured-insights/diversity-and-inclusion/seven-charts-that-show-covid-19s-impact-on-womens-employment# (accessed on 5 June 2021).

While many subjects reported significant sources of stress, the most significant sources of stress were social isolation, loss of school opportunities, and loss of work. Factors directly related to the pandemic, such as illness or death, were present as stressors in the study but not dominant as sources of trauma.

This study has significant limitations, chief among them the convenience sample of university students and the relatively small sample size. The location of this sample may be particularly significant as well, as Massachusetts did not experience the pandemic as severely as some other locations in the United States. In addition, the study was retrospective, as subjects were surveyed between January and April 2021, but asked to recall events and feelings from the past 9 to 12 months. Still, findings here mirror those of larger studies that have found increases in anxiety and depression among children during the coronavirus pandemic. Research in Europe, published in 2021, found that anxiety and depression had increased among children in Italy and Spain, and was associated with parental stress [3]. Research in Brazil showed similar increases. A study of more than 5000 children in China found that increased anxiety and depression following lockdowns was associated with online school problems, insomnia, and family problems during lockdown [4]. The current study adds to this literature by comparatively measuring increases in bullying, fighting, cyberbullying, sexting, and anxiety and depression, and finding that the rise in anxiety and depression dwarfs increases in these other social problems. 

Still, resiliency among subjects varied, with this study finding that females and LGBTQ subjects were more vulnerable to both cyberbullying and anxiety and depression. The increased vulnerability of LGBTQ subjects has been noted in other pandemic research. A yet-to-be-published study in Canada is reporting higher levels of cyberbullying experienced by these youth. Available online: https://www.ctvnews.ca/canada/as-the-pandemic-forces-us-online-lgbtq2s-teens-deal-with-cyberbullying-1.5430945 (accessed on 3 June 2021)). Similarly, an interesting study in India suggested that some factors that pre-pandemic were related to cyberbullying (such as time spent online) became less significant during lockdowns, and that other factors, such as LGBTQ status, became more important in predicting involvement in cyberbullying [5].

Psychological recovery from this pandemic will likely involve, for most children, a return to a more predictable and sociable society, where play and learning take place in person, with peers. A possible loss of social skills over the past year may necessitate particular attention to social problems between children, such as bullying and cyberbullying. In addition, adults should be aware that children are likely to cope with anxiety differently; for example, some may feel compelled to discuss their feelings and experiences, while others may want to avoid such conversations. Techniques such as deep breathing or relaxation exercises may be helpful, along with professional referrals, when appropriate. 

## Data Availability

Data supporting reported results can be found at the Massachusetts Aggression Reduction Center at Bridgewater State University.
